# Prediction of in-hospital mortality and admission to ICU using vital signs - a study of Early Warning Score as an alternative to traditional triage

**DOI:** 10.1186/1757-7241-21-S2-A40

**Published:** 2013-09-09

**Authors:** Momo Menna Illum Vendler, Tobias Thostrup Andersen, Charlotte Barfod, Jakob Lundager Forberg

**Affiliations:** 1 University of Copenhagen, Denmark; 2 Emergency Department, Nordsjællands Hospital, Denmark

## Background

Triage of patients in the Emergency Department includes scoring of vital parameters. The objective of this study was to compare two such triage systems for assessing vital parameters - a single-parameter system, T-vital, as used in Danish Emergency Process Triage, and a multiple-parameter system, T-EWS, which we based on Early Warning Score (EWS) - and correlate the triage scores to in-hospital mortality and admission to ICU. Studies examining EWS in triage are currently limited in number.

## Methods

Using data from the Acute Admission Database of Nordsjællands Hospital (n = 6164 admissions), we calculated and stratified EWS into four T-EWS colour codes (red, orange, yellow, and green), testing different stratifications' correlation to in-hospital mortality and admission to ICU. Afterwards, we compared the ability of the chosen T-EWS and T-vital to predict patients at risk (red and orange category) of in-hospital mortality or admission to ICU. The data were analysed using area under the receiver operating curve (AUROC), sensitivity, specificity, overtriage, undertriage, and diagnostic rates.

## Results

T-vital allocated 10.6% of patients to the orange or red category, whereas T-EWS allocated 5.8% to these categories. There was no significant difference in the ability of T-EWS to predict in-hospital mortality compared to T-vital (AUROC (95% CI): T-EWS = 0.74 (0.70-0.79); T-vital = 0.76 (0.72-0.80)). Likewise, there was no significant difference in prediction of ICU admission (AUROC (95% CI): T-EWS = 0.76 (0.70-0.81); T-vital = 0.73 (0.67-0.79)). The specificity (95% CI) of T-EWS compared to T-vital was higher for both in-hospital mortality (0.95 (0.94-0.95) and 0.90 (0.90-0.91), respectively) and for admission to ICU (0.95 (0.94-0.95) and 0.90 (0.89-0.91), respectively). There was a trend of higher sensitivity of T-vital, and no difference in overtriage, undertriage or diagnostic rates.

## Conclusion

The two triage systems are largely similar in their ability to discriminate patients at high risk of in-hospital mortality or admission to ICU. However, T-vital's larger proportion of orange and red patients might yield a larger workload in the Emergency Department. Replacement of T-vital with T-EWS could be considered, as EWS is already in use as a monitoring tool after triage, but more studies are needed for further clarification.

